# A case of flank pain caused by ureteral intussusception accompanied with ureteral polyp

**DOI:** 10.1186/s12882-020-01904-8

**Published:** 2020-07-01

**Authors:** Yang Dong, Wen-da Zhang, Tao Fan, Lin Hao, Jia-he Zhou, Wei-ming Ma, Cong-hui Han

**Affiliations:** Department of Urology, Xuzhou Central Hospital Affiliated to Xuzhou Medical University, Jiefang South Road, No. 199, Xuzhou, Jiangsu China

**Keywords:** Ureteral intussusception, Ureteral polyp, Ureteroscopy

## Abstract

**Background:**

Ureteral intussusception, a rarely reported unique condition, occurs primarily as a complication of ureteric tumours.

**Case presentation:**

We present a case of ureteral intussusception accompanied with a large ureteral polyp periodically protruding into the bladder cavity occurring in a 56-year-old man who experienced vague flank pain and intermittent haematuria. The patient was successfully treated by ureteroscopic cauterization combined with partial ureterectomy with reanastomosis.

**Conclusions:**

This is the first report that describes polyp-related ureteral intussusception using comprehensive and representative ureteroscopic images and video. Our findings suggest that ureteroscopy is vital for diagnosis. Extensive biopsies through ureteroscopy are less invasive, and make it easier to exclude the presence of ureteral malignancies. Ureteroscopic resection of the whole polyp with its stalk and intussusceptum using Holmium: YAG laser did not seem viable in this case. However, cauterization of partial polyp tissues followed by open surgery for segmental resection of the ureter with reanastomosis is helpful in controlling such patient well-being.

## Background

Ureteral intussusception is a rarely reported unique condition, in which the proximal ureteral wall telescopes into the distal lumen. It often develops slowly and occurs secondary to ureteral neoplasms; however, it is occasionally caused by calculi or endoscopic surgical procedures. Due to the lack of awareness regarding this condition, ureteral intussusception is often unsuspected and misdiagnosed. Here, we present a case of ureteral intussusception accompanied with ureteral polyp and, to our knowledge, this is the first report that provides comprehensive and representative ureteroscopic images and video depicting the case.

## Case presentation

**Fig. 1:**
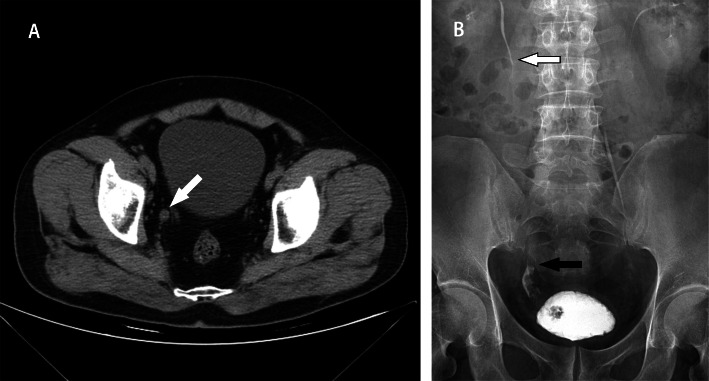
Computed tomography and intravenous urogram findings (**a**) Computed tomography shows an enlargement in the inferior part of the right ureter with a suspected solid mass; (**b**) Intravenous urogram shows a “sponge-like” filling defect in the right distal dilated ureteral lumen (black arrow) without dilation of the upper urinary tract and renal pelvis (white arrow), and an oval-shaped filling defect of the ureteral orifice inside the bladder

A 56-year-old man presented with a 1-week history of vague pain in the right flank and suprapubic region, and intermittent haematuria. He recalled having similar mild attacks periodically 2 years before that remitted without treatment. He denied any significant medical history, the habit of smoking and explosion to any solvents or chemicals. Bladder neoplasm was initially suspected by outpatient colour Doppler ultrasonography and cystoscopy at a local hospital 3 days prior to admission. No abnormalities were observed upon physical examination, serological examination, and urine cytology after admission. Computed tomography (CT) revealed an enlargement in the inferior part of the right ureter with a suspected solid mass (Fig. 1a). Further intravenous urogram (IVU) revealed a “sponge-like” filling defect in the right lower enlarged ureteral lumen and right ureteral orifice inside the bladder, but no dilation of the upper urinary tract and renal pelvis (Fig. 1b). Ureteral tumour was detected preoperatively.

**Fig. 2:**
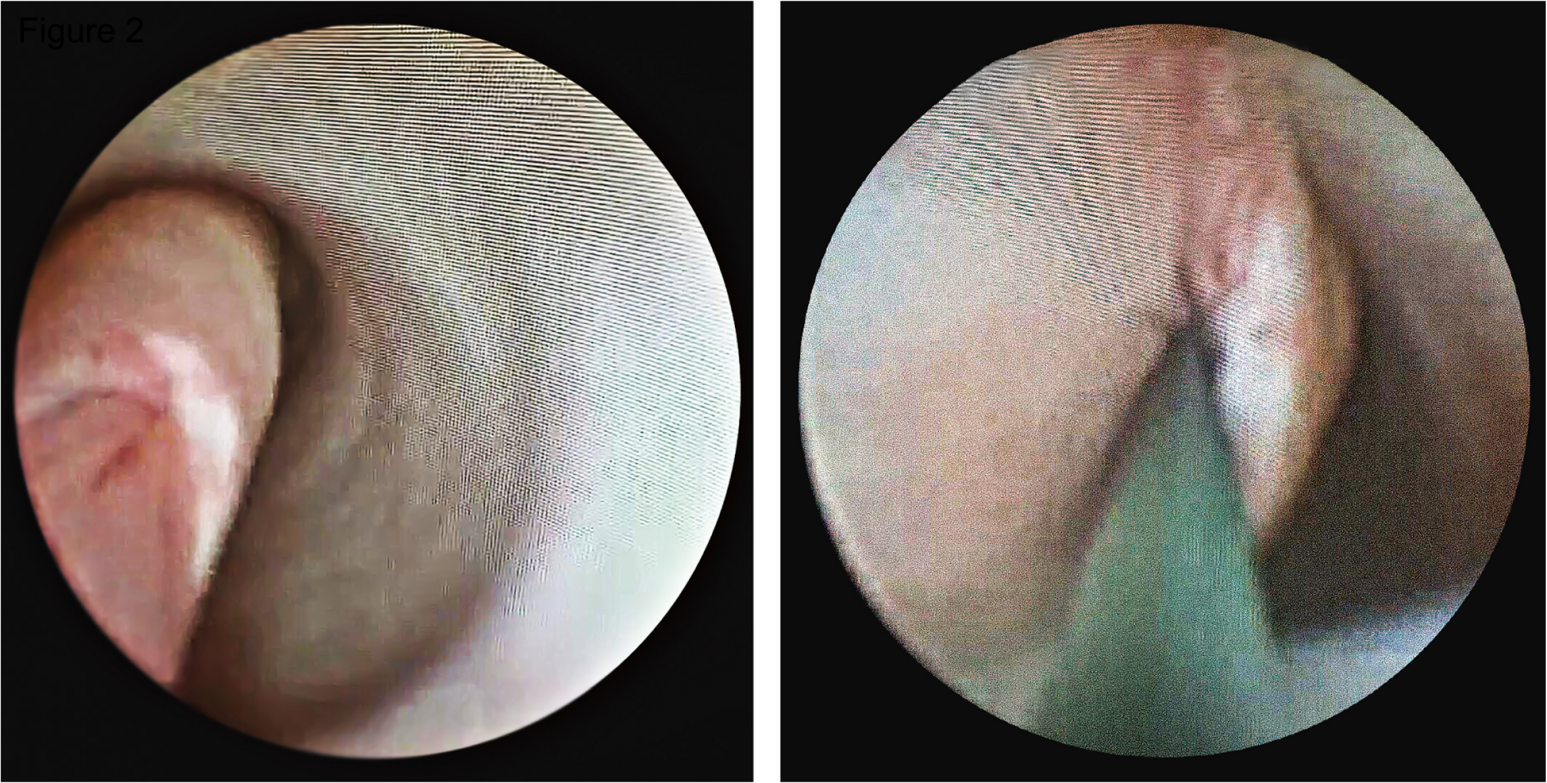
Ureteroscopic view. Ureteroscopy showing an intussuscepted ureter protruding into the lumen of the adjacent segment (**a**); A wire was passed to the renal pelvis through the lumen of the intussusceptum (**b**)

Ureteroscopy revealed that the right lower ureteral lumen was occupied by multiple white-grey polypoid tumours, floating in the ureter that periodically moved in and out of the bladder through the ureteral orifice. Biopsy demonstrated an inflammatory polyp. This polyp together with terminal lobulations was approximately 7.0-cm in length containing a large pedicle that originated from the tip of the proximal ureter. The ureteral lumen terminated at the base of the polyp, but a ureteral orifice was observed in the centre of the proximal pedicle (Fig. 2a). After exploring the interior of the pedicle by ureteroscopy (Fig. 2b), we confirmed that the upper partial pedicle was an intussuscepted segment of the ureter approximately 2.5-cm in length and averaging 8.0-mm in diameter (supplementary video). The patient was treated by ureteroscopic cauterization of the partial polyp at the slimmest point of the pedicle followed by open surgery exploration. A fusiform was found in the lower dilated thick-walled ureter and palpation revealed a firm and mobile tumour. Finally, right ureteral partial resection with reanastomosis was performed to contain the pedicle stalk and ureteral segment. Postoperative recovery was uneventful, and pathological examination confirmed a fibroepithelial polyp. The double-J stent was removed 6 weeks after the operation. A CT scan of the abdomen performed 3 months post-surgery indicated neither uronephrosis nor any signs of polyp recurrence.

**Additional file 1: Supplementary video.** A video recorded during ureteroscopy showing the characteristics of the intussuscepted segment of the ureter in the dilated ureteral lumen.

## Discussion and conclusions

To identify relevant studies up to May 2020, the PubMed database was searched using the terms (“ureteral” [All Fields] OR “ureter” [All Fields] OR “ureter” [MeSH Terms]) AND (“intussusception” [All Fields] OR “intussusception” [MeSH Terms]). We retrieved 101 studies, of which 28 (30 cases) that published from 1937 to 2019 were finally selected after review (Table 1). Of these, 25 cases were related to ureteral neoplasms, including 15 secondary to polyps, and 10 secondary to malignant tumours. The remaining five cases included one ureteral calculus-related case, and four cases of iatrogenic complication occurring in surgical procedures.

**Table 1:** Review of previously published cases of ureteral intussusception

Author	Year	Age/Sex	Number	Aetiology	Lesion Location	Location of pain	Hematuria	Hydronephrosis	NCCT	CECT	CTU	IVU	Treatment
**Dix** [1]	1937	54/M	1	Papilloma	L /Pe-ureter	No	Yes	Yes	N/A	N/A	N/A	No visualization of left ureter	Nephrouretectomy
**Morley** et al. [2]	1952	19/M	1	Fibrous ureteral polyp	R /Pe-ureter	Right flank	No	Yes	N/A	N/A	N/A	The middle ureter is dilated with filling defects, the lower ureter appears normal	Nephrouretectomy (The length of the involved ureter is too long to permit reanastomosis)
**Bonomini** et al. [3]	1963	20/M	1	Benign pedunculated polyp	L /Pe-ureter	Left flank	Yes	Yes	N/A	N/A	N/A	The middle third of the ureter is dilated and occupied with defects of filling	Segmental resection of the ureter with reanastomosis
**Gerdes** et al. [4]	1966	34/F	1	Angiofibromatous polyp	N/A	Diffuse abdomen	Yes	No	N/A	N/A	N/A	N/A	N/A
**Mazer** et al. [5]	1979	66/M	1	TCC	R /Pe-ureter	No	Yes	Yes	N/A	N/A	N/A	A “bell-shaped” filling defect	Local excision of the tumour was performed with reconstruction of the ureter
**Fiorelli** et al. [6]	1981	25/F	1	Fibroepithelial polyp	L /UPJ	Left flank	No	Yes	N/A	N/A	N/A	Ureteral defect and easy flow of contrast medium around it are barely visible.	Resection of the pyeloureteral junction that was shoved into distal ureter after lysis of outer fibrous band was performed followed by an Anderson-Hynes pyeloplasty
**Vogelzang** et al. [7]	1981	27/M	1	Fibrous ureteral polyp	L /Pe-ureter	Left flank	Yes	Yes	N/A	N/A	N/A	Moderate dilatation in the proximal ureter, no contrast passed into intussusception	Tumor excision by ureterotomy, no detail
**Fukushi** et al. [8]	1983	59/F	1	Inflanmmatory Polyp	L /Pe-ureter	Left flank	Yes	Yes	N/A	N/A	N/A	“Claw of crab -shaped” contrast filling sign	Intussusception was repaired after lysis of the external adhesion of the invaginated region
**Takeuchi** et al. [9]**#**	1984	70/M	3	TCC (×3)	R /Ab-ureter	No	Yes	Yes	N/A	“Concentric sign” in ureter and a large mass in bladder (×1)	N/A	A dilatation in the lower ureter with filling defect of varied size, protruding into bladder	Segmental ureterectomy (×1), combined with partial bladder resection (× 1) or radical cystectomy (× 1)
		71/M			L /Ab-ureter and bladder								
		52/M			R /Ab-ureter								
**Haupert** et al. [10]**#**	1985	N/A	1	Fibroepithelial polyp	N/A	N/A	N/A	N/A	N/A	N/A	N/A	N/A	Segmental resection of the ureter with reanastomosis
**Compton** et al. [11]	1986	50/M	1	TCC	R /Pe-ureter	No	Yes	No	N/A	N/A	N/A	A fusiform dilatation in the mid- ureter	Segmental resection of the ureter with reanastomosis
**Gabriel** et al. [12]	1986	74/M	1	TCC	R /Pe-ureter	Right lower abdomen	No	Yes	N/A	N/A	N/A	A local widening with irregular filling defect proximal to the ureterovesical junction	Nephroureterectomy including a cuff of urinary bladder
**Moretti** et al. [13]	1987	59/M	1	TCC	R /Pe-ureter	No	Yes	Yes	N/A	N/A	N/A	A tubular nonopaque filling defect within dilated lower ureter that contains apparently calcified polyp	Intussusception was reduced after local excision of the tumour in ureter
**Duchek** et al. [14]	1987	24/M	1	Papilloma	R /Pe-ureter	Right flank	Yes	Yes	N/A	N/A	N/A	A local widening in the middle ureter with irregular filling defect	Partial resection of the ureter with reanastomosis
**Radhi** [15]	1992	N/A	1	TCC	N/A	N/A	N/A	N/A	N/A	N/A	N/A	N/A	N/A
**Png** et al. [16]	1995	26/F	1	Fibroepithelial polyp	R /Ab-ureter	Right flank	Yes	Yes	N/A	N/A	N/A	A filling defect in the ureter	Segmental resection of the ureter with reanastomosis
**Bernhard** et al. [17]	1996	45/M	1	Latrogenic 1	L /Pe-ureter	Left flank	N/A	Yes	N/A	N/A	N/A	A filling defect in the mid-ureter	Partial resection of the ureter and construction of a Boari flap
**el Khader** et al. [18]**#**	1997	30/F	1	Latrogenic 2	- /UPJ	N/A	N/A	N/A	N/A	N/A	N/A	N/A	Segmental resection of the ureter with calicoureterostomy
**de La Taille** et al. [19]	1998	59/M	1	TCC	R /Ab-ureter	No	Yes	Yes	A ureteral mass	N/A	N/A	Ureteral fusiform enlargement containing an oval filling defect	Nephrouretectomy
**Liu** et al. [20]	2000	63/M	1	Latrogenic 3	R /Ab-ureter	N/A	Yes	Yes	N/A	N/A	N/A	N/A	The intussusception was recognized at the time and was reduced completely with hydrostatic pressure and performed with use of fluoroscopic guidance and conscious sedation.
**Chiong** et al. [21]	2004	15/M	1	Latrogenic 4	R /UPJ	N/A	N/A	Yes	N/A	N/A	N/A	A “bell-shaped” filling defect at the distal part of the upper ureter	Segmental resection of the obstructing ureter with Culp-De-Weerd pyeloplasty reconstruction
**Xu** et al. [22]	2007	49/M	1	Fibroepithelial polyp	R /Pe-ureter	Right flank	Yes	No	An enlargement in the inferior part of the invaginated ureter	“Concentric sign”	N/A	The wall of the intussuscepted ureter appeared as a cylindrical filling defect in the dilatated ureteral lumen. Invaginated ureteral lumen is filled with contrast material and appeared as a “line” sign in the intussusception	Segmental resection of the ureter with reanastomosis
**Jin** et al. [23]	2011	63/F	1	Massive fibroepithelial polyp	R /Pe-ureter	No	Yes	No	N/A	“Concentric sign”	A filling defect in lower enlarged ureter	A “sponge-like” filling defect in lower enlarged ureteral segment and an “oval-shaped” filling defect of the ureteral orifice inside the bladder	Segmental resection of the ureter with reanastomosis via a laparoscopic approach
**Hasegawa** et al. [24]	2011	39/F	1	Fibroepithelial Polyp	R /Pe-ureter	Right flank	No	No	A ureteral mass	N/A	N/A	A “tongue-like” filling defect in the ureter	Segmental resection of the ureter with reanastomosis via a retroperitoneoscopic approach
**Chao** et al. [25]	2012	64/M	1	TCC	R /Pe-ureter	Epigastric	No	Yes	A ureteral mass	“Bull’s-eye sign”	A “stalk-of-corn” appearance on coronal and sagittal imaging	N/A	Nephrouretectomy
**Sewell** et al. [26]	2015	70/M	1	Calculus	L /Pe-ureter	No	No	Yes	A 8 mm calculus	N/A	N/A	A “goblet sign” contrast filling on retrograde pyelography	Ureteropyeloscopic lithotripsy with laser
**Suzuki** et al. [27]	2015	67/M	1	Fibroepithelial polyp	R /Pe-ureter	No	Yes	Yes	N/A	“Concentric sign”	A linear contrast in the invaginated proximal ureter	Invaginated ureteral lumen is filled with contrast material and appeared as a “line-shaped” sign in the intussusception	Segmental resection of the ureter with reanastomosis
**Hajji** et al. [28]	2019	42/M	1	Fibroepithelial polyp	L /Ab-ureter	Right abdomen	No	No	N/A	A ureteral mass protruding into bladder	N/A	A “bell-shaped” filling defect and a “V-shaped” ureter filled with contrast in the upstream from the mass	Resection of the polyp with its stalk by ureteroscopic electrocauterisation
**This case**	2020	56/M	1	Fibroepithelial polyp	R/Ab-ureter	Right flank and lower abdomen	Yes	No	A ureteral mass	N/A	N/A	A “sponge-like” filling defect in lower enlarged ureteral lumen and ureteral orifice inside the bladder	Ureteroscopic cauterization of partial polyp followed by open surgery for segmental resection of the ureter with reanastomosis

Note: #: only English abstract available, *NCCT* non-contrast CT, *CECT* contrast-enhanced CT (on axial imaging), *CTU* CT urography; Ab-ureter: abdominal part of ureter, *Pe-ureter* pelvic part of ureter, *UPJ* ureteropelvic junction, *L* left, *R* right, *TCC* Transitional cell carcinoma, *N/A* not applicable

**Latrogenic 1:** secondary to ureteroscopy; **Latrogenic 2:** secondary to double CH endoprosthesis; **Latrogenic 3;** secondary to percutaneous nephrostomy catheter exchange (retrograde intussusception); **Latrogenic 4**: secondary to percutaneous endopyelotomy for UPJ obstruction

**Fig. 3:**
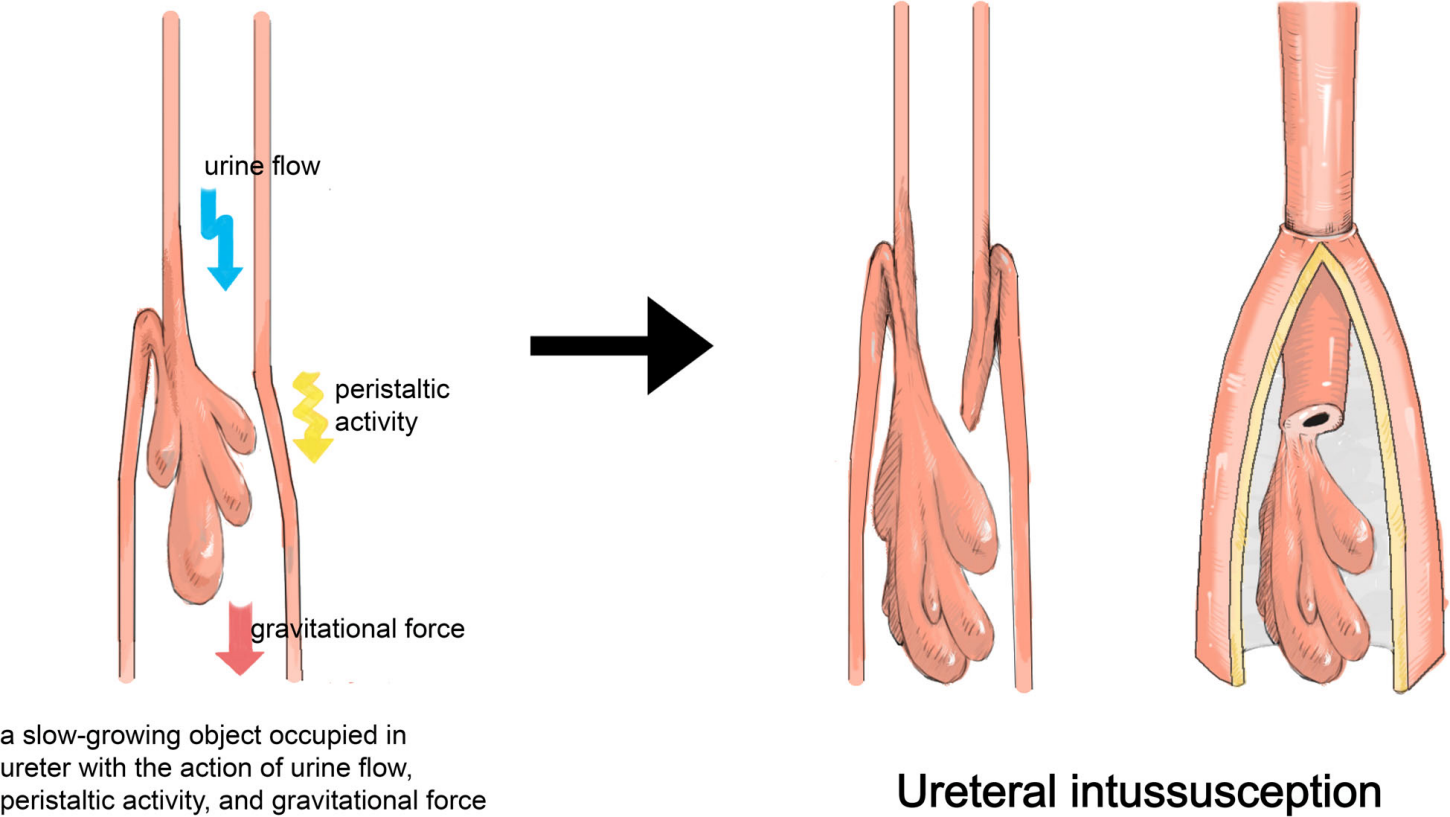
Artist’s schematic line drawing representative of the underlying pathological process

Ureteral intussusception does not occur spontaneously, largely because of the small ratio between ureteral wall thickness and lumen calibre and the limited range of mobility of the ureter itself, which prevents ureter invagination [25]. However, when a slow-growing object, typically a benign tumour, occupies the ureter, it draws down the proximal ureter, enters the distal dilated ureter by peristaltic activity, urine flow, and gravitational force, and sporadically leads to ureteral intussusception [6, 8, 19] (Fig. 3). A review of previously reported cases revealed that intussusception is usually antegrade and more prevalent in men on the right side. The common manifestations in patients include a history of intermittent haematuria and repeated flank pain [25, 26], but malignant tumour-related ureteral intussusception is often asymptomatic and occurs uniformly in patients aged ≥50 years [25]. Some scholars proposed the potential risk of ureteral ischemia because of the possible deficiency of the blood supply to the intussusceptum [28]. However, no evidence of regional ureteral ischemic necrosis associated with ureteral intussusception has been reported previously, which is compatible with the characteristic sluggish changes underlying intussusception.

Ureteral intussusception is characterized by distinct features, especially on IVU and contrast CT images that have great diagnostic value. In primary lesions, ureteral intussusception often presents a “bell-shaped [5, 21, 28]”, “tongue-like [24] “or “sponge-like [23] “filling defect in an enlarged ureteral segment, with or without hydronephrosis. The intussuscepted segment filled with contrast material appears as a “line” sign [22], and the invaginated ureteral lumen appears as a “V-shaped” sign [28], also described as “claw of crab -shaped” sign in the upstream from the mass [8]. Non-contrast CT scan is helpful for revealing calculus and solid ureteral masses but can hardly detect ureteral intussusception. In contrast-enhanced CT images, contrast material opacifies both intussusceptum and distal intussuscipiens, forming a “concentric sign” [9, 22, 23, 27] or “bull’s-eye sign” [25] on axial imaging, and a “stalk-of-corn” appearance on coronal and sagittal imaging [25]. Ureteroscopy and intraoperative biopsy enable definitive diagnosis and are capable of distinguishing between benign or malignant masses. Whereas adequate preoperative imaging examinations can clearly elucidate the extent of the related tumour, which is crucial in presurgical planning. Intussusception accompanied with polyps periodically protruding into the bladder is extremely rare with only three reported cases to our knowledge [23, 24, 28], and could easily be misdiagnosed as bladder tumours by ultrasonography.

In this case, partial lobulated polyps were cauterized using Holmium: YAG laser ureteroscopically, followed by open segmental resection of the ureter with reanastomosis. In the literatures, except for one case reported by F Hajji et al. which was managed by resection of the whole polyp containing the stalk by ureteroscopic electrocauterization and got a following automatic resolution of transient intussusception [28], almost all other cases of benign ureteral tumors were finally managed by surgical resection of the involved ureter with reanastomosis by open or laparoscopic approach [23, 24]. We agree that ureteroscopic cauterization is an effective and minimally invasive treatment for the management of small isolated ureteral polyps with mild and transient ureteral intussusception. However, for patients suffering from stable intussusception complicated with large polyps, it is difficult to resect ureteral lesions completely by ureteroscopic cauterization alone. Besides, because of the limited working space and the dissatisfied laser accuracy ureteroscopically, the pursuit of a perfect excision of intussusceptum will also increase the risk of ureteral perforations, postoperative lesion recurrences, and ureteral strictures. Moreover, James Sewell et al. reported the only case of ureteral calculus-related intussusception, which was treated by ureteropyeloscopic lithotripsy with Holmium: YAG laser [26]. In that case, due to the loose and short range of the intussusceptum, ureteral intussusception resolved automatically following clearance of the calculi [26]. Biopsies of the tumour and intussuscepted ureter via transurethral ureteroscopy would help rule out malignancy and signs of ischemia. The treatment approach should be altered in accordance with the associated primary lesion, the size and location of the polyp, and the occurrence of hydronephrosis [28]. Once histological examination confirms the presence of a ureteral malignant tumour, hemiuridectomy for the urinary tract is required. Ureteral intussusception owing to benign polyps should be treated by local excision of the polyp and reconstruction of the ureter to improve ureteral patency.

In conclusion, by reviewing prior cases and presenting a typical ureteroscopic observation, we hope to increase clinical awareness to this unique condition. Ureteroscopy is vital for diagnosis as it can offer a comprehensive observation to ensure the location and size of the lesions. Extensive biopsies through ureteroscopy are necessary and should be recommended, by which excluding the presence of ureteral malignancies seems to be easier and less invasive. Based on that, treatment option as ureteroscopic cauterization, or in combination with open or laparoscopic surgical resection of segmental ureter can be formulated to benefit such patients.

## Data Availability

All data generated or analysed during this study are included in this published article.

## Abbreviations

CT: Computed tomography
IVU: intravenous urogram
